# A systematic review and functional *in-silico* analysis of genes and variants associated with amyotrophic lateral sclerosis

**DOI:** 10.3389/fnins.2025.1598336

**Published:** 2025-06-16

**Authors:** Carlos A. Arreola-Aldape, Jose A. Moran-Guerrero, Guillermo K. Pons-Monnier, Rogelio E. Flores-Salcido, Emmanuel Martinez-Ledesma, Luis M. Ruiz-Manriquez, K. Rebeca Razo-Alvarez, Daniela Mares-Custodio, Pablo J. Avalos-Montes, Jose A. Figueroa-Sanchez, Rocio Ortiz-Lopez, Hector R. Martínez, Raquel Cuevas-Diaz Duran

**Affiliations:** ^1^Tecnologico de Monterrey, Escuela de Medicina y Ciencias de la Salud, Monterrey, Mexico; ^2^Instituto de Neurología y Neurocirugía, Centro Médico Zambrano Hellion, TecSalud, San Pedro Garza Garcia, Mexico; ^3^Department of Neurology, University of Wisconsin School of Medicine and Public Health, Madison, WI, United States; ^4^Tecnologico de Monterrey, Institute for Obesity Research, Monterrey, Mexico; ^5^Tecnologico de Monterrey, Proyecto oriGen, Monterrey, Mexico

**Keywords:** Amyotrophic Lateral Sclerosis, systematic review, genes, variants, mutations

## Abstract

**Introduction:**

Amyotrophic lateral sclerosis (ALS) is a fatal progressive neurodegenerative disease characterized by the deterioration of upper and lower motor neurons. Affected patients experience progressive muscle weakness, including difficulty in swallowing and breathing; being respiratory failure the main cause of death. However, there is considerable phenotypic heterogeneity, and its diagnosis is based on clinical criteria. Moreover, most ALS cases remain unexplained, suggesting a complex genetic background.

**Methods:**

To better understand the molecular mechanisms underlying ALS, we comprehensively analyzed, filtered and classified genes from 4,293 abstracts retrieved from PubMed, 7,343 variants from ClinVar, and 33 study accessions from GWAS catalog. To address the importance of ALS-associated genes and variants, we performed diverse bioinformatic analyses, including gene set enrichment, drug-gene interactions, and differential gene expression analysis using public databases.

**Results:**

Our analysis yielded a catalog of 300 genes with 479 ALS-associated variants. Most of these genes and variants are found in coding regions and their proteins are allocated to the cytoplasm and the nucleus, underscoring the relevance of toxic protein aggregates. Moreover, protein-coding genes enriched ALS-specific pathways, for example spasticity, dysarthria and dyspnea. ALS-associated genes are targeted by commonly used drugs, including Riluzole and Edaravone, and by the recently approved antisense oligonucleotide therapy (Tofersen). Moreover, we observed transcriptional dysregulation of ALS-associated genes in peripheral blood mononuclear cell and postmortem cortex samples.

**Conclusion:**

Overall, this ALS catalog can serve as a foundational tool for advancing early diagnosis, identifying biomarkers, and developing personalized therapeutic strategies.

## 1 Introduction

Amyotrophic Lateral Sclerosis (ALS) is a devastating, progressive neurodegenerative disorder whose hallmark is the degeneration of both upper and lower motor neurons in the cerebral cortex, brainstem, and spinal cord (Swinnen and Robberecht, 2014; Galvin et al., 2017; Hardiman et al., 2017). ALS is one of the most common adult motor neuron diseases, representing a significant socioeconomic burden (Logroscino et al., 2018). Although affected by ethnicity, the global incidence of ALS is approximately 2 cases per 100,000 person-years with a prevalence of 6-9 per 100,000 individuals (Martínez et al., 2011; Mehta et al., 2018; Longinetti and Fang, 2019; Brown et al., 2021; Wolfson et al., 2023). The number of ALS cases is rapidly increasing, mainly due to population aging. Furthermore, projections based on a meta-analysis of reported cases show that the number of ALS patients worldwide will likely increase to 375,000 in 2040, representing a 69% rise (Arthur et al., 2016). Moreover, the cumulative lifetime risk for developing ALS is estimated to be 1:350 in men and 1:400 in women (Ryan et al., 2019). The age of onset is approximately between 50 and 75 years, and even though the rate of disease progression is variable, most patients die of respiratory muscle failure within 2-3 years of symptom onset (Masrori and Van Damme, 2020).

There is considerable variation in the phenotypic expression of ALS including the onset site, age of onset, type and degree of motor neuron involvement, disease progression, symptom severity, and survival time (Tard et al., 2017; Feldman et al., 2022). Approximately 70% of ALS patients present a spinal onset characterized by muscle weakness of the limbs, 30% present with a bulbar onset distinguished by dysarthria, dysphagia, and dysphonia, and a minority (3-5%) have a respiratory or cognitive onset (Tard et al., 2017; Masrori and Van Damme, 2020; Feldman et al., 2022) The bulbar and respiratory onset are generally associated with a poor prognosis (Kiernan et al., 2011); the former being more common in women (Salameh et al., 2015). Despite being predominantly considered a motor disease, ALS patients also present cognitive and behavioral changes (Beeldman et al., 2020; Pender et al., 2020). Thus, there is a molecular overlap between ALS and Frontotemporal dementia (FTD) since approximately 15% of ALS patients meet FTD diagnostic criteria (Ringholz et al., 2005; Pender et al., 2020). Due to the heterogeneous presentation of ALS, diagnostic criteria are available, including the revised El Escorial criteria (Brooks et al., 2000), the Awaji Shima criteria (de Carvalho et al., 2008), and recently, the simplified Gold Coast criteria (Shefner et al., 2020). However, there is no definite test for ALS diagnosis. Instead, a clinical investigation, consisting of blood tests, imaging of the brain and spine, and neurophysiological evaluations, is performed to exclude mimic disorders (Turner and Talbot, 2013). Thus, there is an urgent need for accurate diagnostic criteria for ALS to reduce the diagnostic delay (∼1 year after disease onset), granting early treatment initiation and enabling an improved prognosis.

ALS is mainly considered a sporadic disease (sALS) because 80–90% of the cases depict no known genetic mutation. In contrast, 10% of ALS patients have a family history of disease with an autosomal dominant inheritance pattern (fALS). Intriguingly, mutations in merely more than 30 genes have been identified as causative or conferring an increased risk of the development of ALS, explaining 70% of fALS and only 15% of sALS (Renton et al., 2014; Chia et al., 2018; Mead et al., 2023). However, the heritability of ALS, both sporadic and familial, has been estimated to be approximately 50% (Ryan et al., 2019; Trabjerg et al., 2020). To explain the heritability of ALS, genome-wide association studies (GWAS) and next-generation sequencing technologies have led to the identification of several ALS risk loci (Van Rheenen et al., 2016; Nicolas et al., 2018; van Rheenen et al., 2021). Nevertheless, these changes explain less than 10% of ALS cases, suggesting that a large number of ALS risk genes are still unknown. Known risk genes converge on common biological pathways such as oxidative stress, mitochondrial function dysregulation, protein homeostasis, RNA processing, DNA damage, and excitotoxicity.

Interestingly, mutations in four genes account for 70% of fALS cases, namely, C9orf72, TARDBP, SOD1, and FUS (Chiò et al., 2014; Chia et al., 2018). However, the genetic architecture of ALS is complex because a minority of patients exhibit a monogenic inheritance, while the majority have an oligogenic pattern characterized by the inheritance of mutations in several genes. Furthermore, researchers have identified a shared polygenic risk of ALS with traits and conditions including smoking, physical activity, cognitive performance, and educational attainment (Bandres-Ciga et al., 2019). In addition, unprecedented recent research combining transcriptomic and epigenetic profiling of motor neurons, GWAS statistics, and machine learning methods identified 690 potential ALS-associated genes, representing a 5-fold increase in the heritability of the disease (Zhang et al., 2022).

Mounting evidence demonstrates the contribution of numerous genetic variants to the risk of ALS in different cohorts. However, an updated and comprehensive collection of ALS-associated genes and variants is needed. To this aim, we performed a systematic review analyzing 4,293 abstracts from PubMed, 7,343 variants from ClinVar, and 33 study accessions from GWAS catalog. Furthermore, we performed numerous functional analyses to verify that our list of genes was associated with ALS.

## 2 Materials and methods

### 2.1 Systematic literature search

**FIGURE 1:**
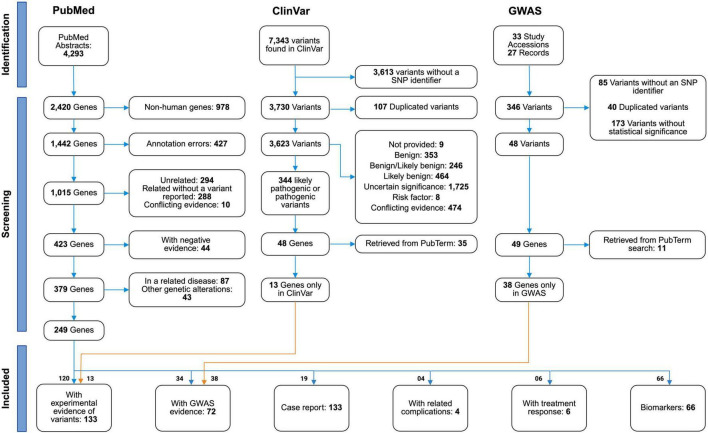
Flowchart of literature selection. PRISMA flowchart summarizing the steps of the screening process with details regarding the number of publications retrieved by the initial query, the number of reports excluded at each screening step, and the number of studies selected for the review from PubMed, ClinVar, and GWAS.

A systematic search for relevant articles was performed in February 2023 using PubTerm (Garcia-Pelaez et al., 2019); a curation and annotation webtool. Our search strategy included two main terms and their synonyms: amyotrophic lateral sclerosis and variants. Articles were restricted to original research papers and to humans. Thus, the following query was used: (“Amyotrophic Lateral Sclerosis”[TIAB] OR “Lou Gehrig”*[TIAB] OR “ALS”[TIAB]) AND (mutation*[TIAB] OR polymorphism*[TIAB] OR variant*[TIAB] OR SNP*[TIAB]) AND English[Language] NOT review[Publication Type] NOT mouse[TIAB] NOT mice[TIAB] NOT animal*[TIAB] to retrieve records from PubMed database (Supplementary Table 1). The process for record selection followed the Preferred Reporting Items for Systematic Review and Meta-Analysis (PRISMA) 2020 guidelines (Page et al., 2021), as depicted in Figure 1. Our systematic literature search was complemented with searches for ALS-associated variants performed in ClinVar and GWAS databases as will be described below.

### 2.2 Curation and gene categorization

Using the PubTerm annotation web tool, we organized retrieved articles by gene, and we defined criteria for filtering records (Figure 1). Records corresponding to non-human genes or to genes resulting from annotation or nomenclature errors were eliminated. The remaining records underwent a scrutinous revision through a manual curation of the title, abstract, and in some cases, the complete manuscript. At least two authors performed the manual curation. Genes in the following exclusion categories were filtered out: (1) unrelated, (2) not reported, (3) conflicting evidence, (4) negative evidence, (5) related disease, (6) other genetic alteration. Genes were “unrelated” when they were not associated with ALS or any other neurological disorder. Additionally, “not reported” genes were those that were related to ALS, but their variants were not described. Moreover, genes were annotated as “conflicting evidence” when articles disagreed on the association of the gene variant with ALS risk. Genes were noted as “negative evidence” when researchers reported no association between the gene variant and ALS. Furthermore, genes with variants found enriched in diseases related to ALS (neurodegenerative, neuromuscular, neurological) were labeled as “related disease.” Finally, genes in which the variant was associated to ALS but there was uncertainty in its locus, or its single-nucleotide polymorphism (SNP) was found in an intronic or intergenic region, were considered as “other genetic alterations.”

Abstracts organized per gene were carefully analyzed until enough evidence was obtained to classify the gene into a relevant inclusion category. We defined six categories of genes with variants associated with ALS: (1) gene variants with experimental evidence of association to ALS, (2) gene variants found in gene exons and with evidence in a GWAS, (3) gene variants described in a case report, (4) gene variants found to be related to ALS complications, (5) gene variants related to treatment response, and (6) genes (with or without variants) that were suggested as biomarkers of ALS. Genes that potentially fit more than one category were assigned to the class with the strongest evidence or the highest number of supporting abstracts. The resulting genes and their categories are included in Supplementary Table 2. For every gene, sentences from abstracts that were critical for assigning a gene’s category, along with the abstracts’ PubMed ID, were annotated in PubTerm’s notes. Annotations for each gene are available in Supplementary Table 3.

### 2.3 Collection of ALS-associated variants

To find additional genes whose variants were not explicitly described in PubMed records, we searched ClinVar, a public database of human variant-phenotype associations (Landrum et al., 2018). We downloaded ALS associated variants from ClinVar website^1^ (Accessed on June 15, 2023) using the search term “Amyotrophic Lateral Sclerosis.” Variants without a defined SNP identifier (dbSNP ID) were filtered out. Variants were assigned a severity score according to their clinical significance and these scores were used for filtering redundant variants (same SNP identifier), keeping unique variants of highest severity (lowest score). Furthermore, only variants with severity scores in the categories “pathogenic” or “likely pathogenic” were used for downstream analysis. A list of genes mapped to variants was compiled and filtered to exclude genes resulting from the PubTerm revision. The remaining genes were classified as having experimental evidence of variants. The list of variants and clinical significance are included in Supplementary Table 4.

To further collect ALS associated variants, we explored the GWAS Catalog (Sollis et al., 2023), a comprehensive database of human genotype-phenotype associations derived from curated GWAS. All association studies related to the “Amyotrophic Lateral Sclerosis” trait and corresponding to the MONDO_0004976 disease ontology were retrieved from the GWAS Catalog webpage^2^ (Accessed on June 15, 2023) and can be found in Supplementary Table 4. Only unique variants with a defined SNP identifier, and with a significance *p*-value less than 5*x* 10^−8^ were considered for downstream analysis. In the cases of redundant variants (same SNP identifier), the smallest *p-*value was considered. A list of genes spanning the remaining GWAS variants was obtained and filtered to exclude genes resulting from the PubTerm search.

A catalog of SNP identifiers was compiled from the PubMed, ClinVar, and GWAS systematic revisions. We extracted genomic information of each variant from either GWAS, ClinVar or through the rentrez R package (Winter, 2017). Variants were classified as intronic, intergenic or exonic according to their genomic location using Homer annotation tool (Heinz et al., 2010) and the hg38 annotation file version 21 downloaded from GENCODE^3^. The ALS variants catalog and relevant genomic information is included in Supplementary Table 5.

### 2.4 *In-silico* determination of gene functions

Genes resulting from our systematic review were classified according to the gene type (protein-coding, non-protein coding and others) specified in the hg38 GENCODE annotation file version 21 downloaded from GENCODE (see text footnote 3). The subcellular location of the molecules coded by each protein-coding gene was then retrieved from the UniProtKB/Swiss-Prot database (Bateman et al., 2023) accessed at https://www.uniprot.org/ on June 15, 2023. The cellular location of the first isoform of genes coding for multiple isoforms was used. Only records with a “reviewed” status and “Homo sapiens” category were retrieved. Additionally, all genes that encode known transcription factors (TF) were further identified using a list of human TFs obtained by combining the AnimalTFDB v4.0 (Shen et al., 2023) and the *The Human Transcription Factors* database (Lambert et al., 2018) downloaded from http://humantfs.ccbr.utoronto.ca/ on June 15, 2023 (gene type, subcellular location data, and TF classification are included in Supplementary Table 6).

### 2.5 Gene set enrichment analysis

We used ALS associated genes to estimate the enrichment of gene sets through a hypergeometric statistical test (phyper R function). We used the msigdbr *R* package to download the Molecular Signatures Database (MSigDB) (Subramanian et al., 2005; Liberzon et al., 2015) and obtained human specific gene sets (hallmark gene sets, Gene Ontologies, Biocarta, Reactome, and KEGG pathways). Gene sets with FDR < 0.05 and at least 5 overlapping genes were considered significantly enriched and are listed in Supplementary Table 7. All protein-coding genes were considered within the gene universe for the hypergeometric test due to their propensity of having variants. GraphPad Prism 9 tool was used to generate Figs depicting the enrichment of significant ALS related gene sets grouped by clinical phenotype and manifestations. Overlaps of genes in significant gene sets related to ALS, cognitive impairment, depression, dementia, and FTD were performed with the InteractiVenn online tool (Heberle et al., 2015).

### 2.6 Comparison with canonical and machine learning-based predicted ALS-associated genes

The compiled set of ALS-associated genes were compared to an independently curated list of ALS genes (*n* = 260) (Eitan et al., 2022) and to a machine learning-derived set (*n* = 690) (Zhang et al., 2022). Lists are included in Supplementary Table 8.

### 2.7 Analysis of drug-gene interactions

A drug-gene interaction analysis was performed using the drug-gene interaction repository (February 2022) from *The Drug Gene Interaction Database* (DGIdb) (Cannon et al., 2024). We compiled drug-gene interactions by using the list of ALS-associated genes as input with default parameters. We downloaded the list of interactions and filtered them for further analysis and visualization using *Cytoscape V3.10* (Shannon et al., 2003). Genes depicting interactions with less than two drugs were filtered out. The full list of interactions obtained is provided in Supplementary Table 9.

### 2.8 Differential gene expression analysis

To further assess the relevance of our list of ALS-associated genes with variants, we performed a differential expression analysis using two recently available high throughput sequencing ALS datasets downloaded from NCBI’s Gene Expression Omnibus (Barrett et al., 2013). We downloaded the FPKM expression matrix of GSE183204 which includes transcriptomic data of peripheral blood mononuclear cells isolated from 18 ALS patients and 12 age and sex-matched healthy controls. In this research, authors stratified ALS patients according to levels of nuclear SOD1 as high and low (Garofalo et al., 2022). Given that high levels of nuclear SOD1 are hypothesized to have a protective mechanism in sALS patients (Garofalo et al., 2022), we compared healthy controls against sALS patients with low nuclear accumulations of SOD1. Similarly, we downloaded the collection GSE124439 composed of transcriptomic datasets obtained from 148 ALS postmortem cortex samples and 17 neurologically healthy controls (Tam et al., 2019). In this research, authors identified 3 distinct molecular ALS subtypes: retrotransposon activation, oxidative stress, and activated glia. Due to their lowest transcriptional heterogeneity, we selected samples classified as activated microglia (ALS-glia) to compare against neurologically healthy controls. Raw count matrixes were downloaded from GEO.

For the first data set (GSE183204) gene expression values were log-transformed and quantile normalized whereas the second data set (GSE124439) was processed using the variance stabilizing transformation (VST) included in DESeq2 (Love et al., 2014) and quantile normalization. For each data set we performed a principal component analysis (PCA) using 1,000 most variable genes according to the median absolute deviation. Outlier samples were identified by plotting the first two principal components and were eliminated from downstream analysis. Focusing only on the list of ALS-associated genes, we performed a differential expression analysis using Limma (Ritchie et al., 2015) and DESeq2 (Love et al., 2014) for the first and second data sets, respectively. Genes with a normalized expression fold-change > 1.1 and a false discovery rate (FDR) < 0.1 were considered differentially expressed. The Gencode v43 human gene annotation file was downloaded^3^ and used. All statistical analysis were performed in the R programming language. Heatmaps of normalized expression matrices depicting differentially expressed genes were constructed (Figure 6). The matrices of normalized counts and differential expression metrics of both public data sets are included in Supplementary Table 10.

## 3 Results

### 3.1 The systematic review identifies and classifies genes with ALS-associated variants

We performed a systematic review using PRISMA’s standards to comprehensively collect all genes with variants associated with ALS. A total of 4,293 abstracts (Supplementary Table 1) matching the PubMed query described were imported into the PubTerm web tool (Garcia-Pelaez et al., 2019). Through automatic annotation, abstracts yielded 2,420 genes that were subsequently filtered eliminating non-human (*n* = 978) and incorrectly annotated genes (*n* = 427). The remaining 1,015 genes were categorized for exclusion through a manual screening of the titles and abstracts of all records associated with each gene. If needed, the complete publications were reviewed for adequate gene categorization. Overall, we found 294 genes that were not related to ALS nor to any other neurological disorder and 288 genes described as associated with ALS but without a variant description. Furthermore, genes related to ALS with variants described were classified as “conflicting evidence” (*n* = 10) or “negative evidence” (*n* = 44) when studies disagreed on the association of a gene variant to ALS or when they reported no association, respectively. The remaining 379 genes were filtered if their variants were associated with ALS-related diseases, (neurodegenerative, neuromuscular, and neurological disorders) (*n* = 87) or if gene variants were found in intronic or intergenic regions (*n* = 43). Overall, our systematic review yielded 249 genes from PubMed with genomic variants related to ALS (Figure 1).

The resulting genes (*n* = 249) were further assigned to inclusion categories according to the type of evidence provided in their related articles. A total of 120 and 34 genes reported variants with experimental or GWAS evidence, respectively, whereas 4 and 6 genes depicted variants related to disease complications and treatment response. Moreover, 19 and 66 genes, were described as having variants found in case reports or suggested as potential biomarkers, respectively. The classification of genes in either exclusion or inclusion categories is depicted in Supplementary Table 2. The critical sentence considered as supporting evidence of classification into inclusion or exclusion categories is included in Supplementary Table 3.

To further collect ALS associated variants, we explored the ClinVar database (Landrum et al., 2018) and the GWAS Catalog (Sollis et al., 2023) using the “Amyotrophic Lateral Sclerosis” search query. We retrieved 7,343 variants from the ClinVar database and filtered 3,613 variants which lacked an SNP identifier yielding 3,730 variants. The remaining variants were assigned a severity score based on their clinical significance. A total of 107 variants were redundant and only those with highest severity (lowest score corresponding to pathogenic or likely pathogenic) were maintained (*n* = 3,623). Interestingly, almost 50% of ALS variants (1,725/3,623) had an uncertain significance and 13% (474/3,623) were classified as “Conflicting interpretations of pathogenicity”, suggesting that more studies associating those genes and their variants to ALS pathogenesis are needed.

We further filtered variants leaving only those with pathogenic and/or likely pathogenic scores, yielding 344 variants mapped to 48 genes (included in Supplementary Table 4). The SNP identifiers of these variants were aggregated to our list of ALS-related SNPs (Supplementary Table 5). We compared the 48 ClinVar genes to the list of ALS genes derived from PubTerm and we found that 35 genes overlapped and were distributed among the inclusion categories of “With experimental evidence of variants” (*n* = 32), “With GWAS evidence” (*n* = 1), and “Case report” (*n* = 2). After filtering these overlapping genes, we obtained 13 genes annotated exclusively in ClinVar (included in Supplementary Table 4) and we assigned them to the “experimental evidence” category.

Similarly, from the GWAS Catalog we retrieved 346 variants reported in 33 different study accessions (27 publications) and matching the defined query (Supplementary Table 4). We found 85 variants without an assigned SNP identifier, 40 redundant variants, and 173 non-significant (*p*-value > 5 ×10^–8^) variants and filtered them out. We found significant variants annotated as intergenic in GWAS Catalog (*n* = 10) that were mapped to more than one gene due to their proximity. In such cases, the same variant was assigned individually to all neighboring genes. Thus, we obtained 58 variants (48 unique ones) that mapped to 49 genes of which 11 were found in our PubTerm search and were either classified as having experimental (*n* = 6) or GWAS evidence (*n* = 5). Subsequently, 38 genes found exclusively in the GWAS Catalog were added to the category of genes with GWAS evidence of variants (Figure 1). Intriguingly, filtered variants were derived from studies based mainly on genome-wide/exome-wide genotyping arrays or targeted genotyping sequencing, potentially overlooking non-coding variants.

**FIGURE 2:**
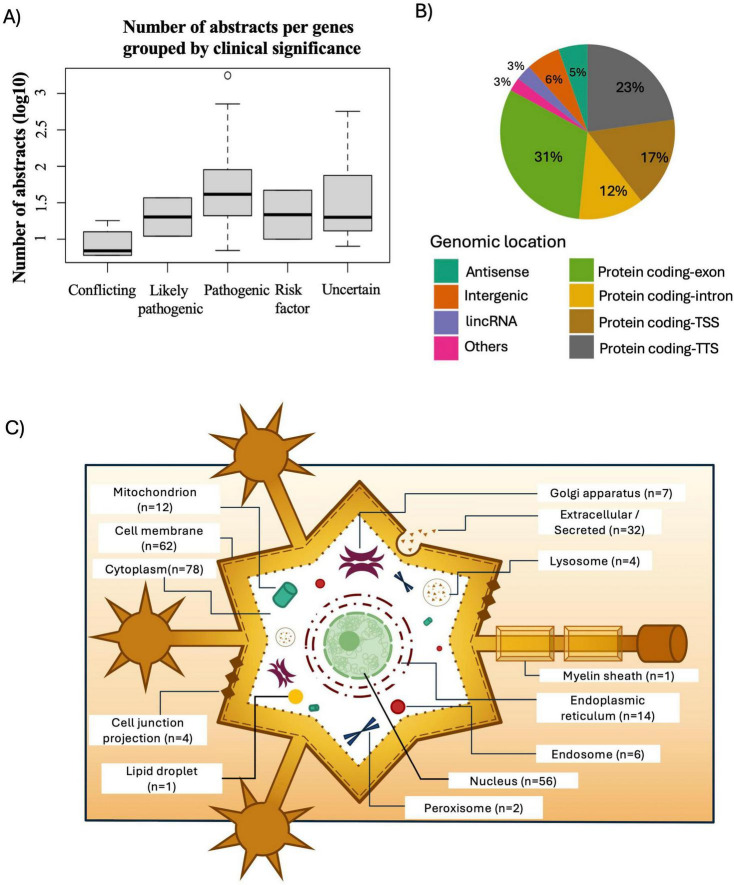
Characterization of variants according to clinical significance, genomic location, and subcellular location of proteins. **(A)** Number of abstracts (log10-transformed scale) found per ALS-associated gene and the clinical significance category of their variants as classified by ClinVar. **(B)** Classification of variants associated with ALS-genes according to their genomic location. **(C)** Schematic representation of the subcellular location of proteins coded by ALS-associated genes.

To evaluate the strength of ALS-associated genes we combined the genes found in all databases, yielding 300 ALS-associated genes (249 PubTerm, 13 ClinVar, 38 GWAS). From these, 71 genes were found to have at least 5 publications associated in the PubTerm/PubMed search (Supplementary Table 4). Interestingly, genes with the highest number of publications are commonly known ALS-associated genes: SOD1, TARDBP, C9orf72, and FUS (Table 1). For each gene, we added its inclusion category, and whether it was found in the Clinvar search after filtering redundant and unidentified variants. Moreover, for each gene found with variants in Clinvar, we added its clinical significance. If a gene had more than one variant, we selected the one with the highest severity. We also labeled genes with significant (*p*-value < 5 × 10^–8^) variants registered in GWAS Catalog. Surprisingly, only 6 genes (with at least 5 publications) were annotated in GWAS catalog as having ALS-associated variants, suggesting that more GWAS studies in diverse ethnicities are needed. We built a boxplot from the combined datasets to determine if there was a correlation between a gene’s clinical significance and the number of abstracts assessed in PubTerm. As shown in Figure 2A, genes with pathogenic clinically significant variants have a higher median number of abstracts/publications associated with ALS. Similarly, the top 20 genes with the highest number of abstracts are all found in Clinvar and have a pathogenic clinical significance (see Table 1).

**TABLE 1:** Top 20 genes with the higher number of abstracts associated with ALS with our search query using PubTerm.

Genes	No. abstracts	Inclusion category	ClinVar	Clinical significance	GWAS
SOD1	1,763	With experimental evidence of variants	Yes	Pathogenic	Yes
TARDBP	717	With experimental evidence of variants	Yes	Pathogenic	No
C9orf72	567	With experimental evidence of variants	Yes	Uncertain significance	Yes
FUS	486	With experimental evidence of variants	Yes	Pathogenic	No
MAPT	118	With experimental evidence of variants	Yes	Pathogenic	No
VCP	112	With experimental evidence of variants	Yes	Pathogenic	No
OPTN	96	With experimental evidence of variants	Yes	Pathogenic	No
SQSTM1	89	With experimental evidence of variants	Yes	Pathogenic	No
ANG	74	With experimental evidence of variants	Yes	Uncertain significance	No
UBQLN2	73	with experimental evidence of variants	Yes	Pathogenic	No
TBK1	71	With experimental evidence of variants	Yes	Pathogenic	Yes
VAPB	69	With experimental evidence of variants	Yes	Pathogenic	No
SETX	53	With experimental evidence of variants	Yes	Pathogenic	No
ALS2	50	With experimental evidence of variants	Yes	Pathogenic	No
ATXN2	46	With experimental evidence of variants	Yes	risk factor	No
PFN1	44	With experimental evidence of variants	Yes	Pathogenic	No
CHCHD 10	37	With experimental evidence of variants	Yes	Pathogenic	No
HNRNPA1	36	With experimental evidence of variants	Yes	Likely pathogenic	No
MATR3	35	With experimental evidence of variants	Yes	Pathogenic	No
KIFSA	27	With GWAS evidence	Yes	Pathogenic	Yes

To analyze the ALS-associated variants found we compiled a list of 479 SNP identifiers mapped to 146 unique genes. Most variants were downloaded from ClinVar (70%), PubMed (16%), and GWAS (11%), however, a minority were shared among PubMed/ClinVar (2%) and PubMed/GWAS (1%) as depicted in Supplementary Figure 1. Furthermore, we extracted genomic information of ALS-associated variants using rentrez R package (Winter, 2017) and Homer annotation tool (Heinz et al., 2010). As observed in Figure 2B, ALS-associated variants are located mainly in protein-coding regions including exons (31%), promoter-transcription start sites (TSS) (17%) and transcription termination sites (TTS) (23%). However, variants are also found in intronic (12%) and intergenic regions (6%) and the remaining (11%) in regulatory regions such as long intergenic RNAs (lincRNAs), antisense lncRNAs, and others (pseudogenes, and small nucleolar RNAs). A catalog of all compiled variants, RS identifiers, mapped genes, genomic location, source database, and region type is found in Supplementary Table 5.

### 3.2 ALS-associated genes code for proteins mainly found in the cytoplasm, cell membrane, and nucleus

To assess the functional relevance of our list of 300 ALS-associated genes, we searched for gene type information in the GENCODE annotation file and found that 275, 11, and 4 genes were classified as protein-coding, lncRNA, and miRNA, respectively. The remaining 10 genes belonged to snRNA or pseudogene categories. Furthermore, we analyzed the subcellular location of ALS-associated proteins using UniProtKB/Swiss Prot database and found that while the majority of proteins are found in the cytoplasm (*n* = 78), cell membrane (*n* = 62) or nucleus (*n* = 39), others are secreted (*n* = 32) or located in the endoplasmic reticulum (*n* = 14) or mitochondrion (*n* = 12), suggesting diverse dysregulated cellular pathways. For example, major ALS-related genes C9orf72, FUS, and TARDBP, are allocated to the nucleus, whereas SOD1, is found in the cytoplasm. Mutations in TARDBP, FUS, C9orf72, and SOD1 may result in toxic protein aggregates in neurons, leading to degeneration in ALS. Interestingly, we found that only 4% (12/300) of ALS-associated genes including CAMTA1, CEBPD, KCNIP3, LHX8, MEF2C, MTF1, RUNX2, SFPQ, SREBF1, TFAM, ZNF704 and ZNF746, are annotated as transcription factors. The subcellular location of ALS-associated proteins is depicted in Figure 2C. Supplementary Table 6 includes gene type, subcellular location, and the classification of transcription factors.

### 3.3 Functional gene set enrichment analysis identifies ALS-relevant gene sets

**FIGURE 3:**
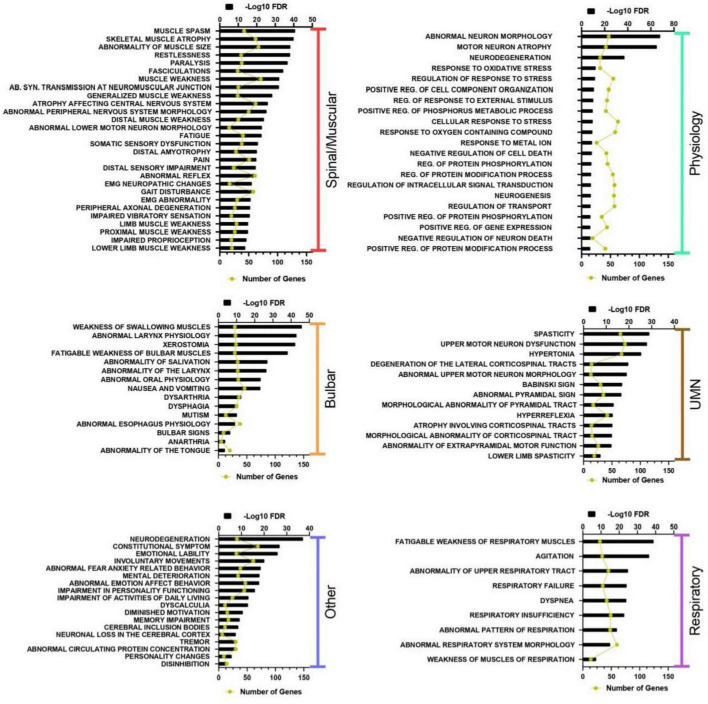
Highly enriched gene sets and ontologies classified. We identified (hypergeometric test with FDR < 0.05 and at least 5 common genes). Enriched gene sets were grouped by relevant physiological categories. Enrichment is indicated as -log10(FDR) in black bars and the number of common genes is represented with the yellow line. AB, Abnormal; UMN, Upper Motor Neuron; REG. Regulation; SYN, Synaptic.

To summarize the biological significance of the ALS-associated genes found, we performed a gene set enrichment analysis. To investigate the most significant gene sets across a variety of domains such as diseases, bioprocesses and cellular functions, we used the GO, Hallmark Gene Sets, Reactome, KEGG pathways and the Biocarta databases. Gene sets were considered enriched with a False Discovery Rate (FDR) < 0.05 and at least 5 overlapping genes. The full list of enriched gene sets is included in Supplementary Table 7. Similar functional terms were manually grouped into panels for comprehensive interpretation and expository purposes as shown in Figure 3. Significant terms that were related to different components of ALS were grouped under spinal/muscular, bulbar, respiratory and upper motor neuron categories. Terms related to biochemical processes, cellular components and metabolism were grouped in a physiology category and common ALS phenotypes and symptoms were classified as “other.”

**FIGURE 4:**
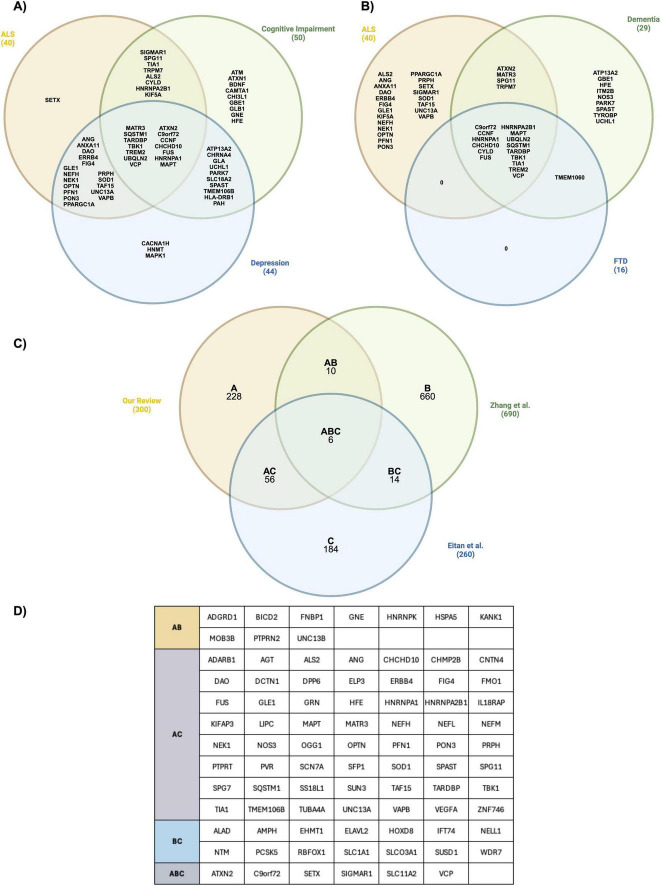
Comparison of ALS-associated genes with disorders depicting similar symptoms and curated/machine-learning ALS lists. Venn diagrams depicting the ALS-associated genes that are common and specific between different enriched human phenotype gene sets. **(A)** ALS, Cognitive Impairment, and Depression. **(B)** ASL, Dementia, and Frontotemporal Dementia (FTD). **(C)** Venn diagram depicting common genes between ALS-associated genes and an independently curated list of ALS genes proposed by Eitan et al. (2022) and a list of genes inferred by Zhang et al. (2022) through a machine learning approach using transcriptomic and epigenomic cell profiling. The list of genes proposed in this Venn diagram are referred to as **A**, while those proposed by Zhang et al. and Eitan et al. are labelled as **B** and **C** respectively. **(D)** List of the common genes found in the comparisons depicted in subsection **(C)**.

As expected, Amyotrophic Lateral Sclerosis was identified as the most significantly enriched disease gene set (−log10 FDR = 66.7). Other significant disease gene sets found included depression, cognitive impairment, frontotemporal dementia and dementia, suggesting that ALS-associated genes are part of a shared genetic background including other neurodegenerative disorders. Figures 4A,B depict the genetic overlap between these disorders. Genes like C9orf72, FUS, VCP, TBK1, CCNF, HNRNPA1, and SQSTM1 among others are common to all diseases. The pairing of ALS-depression shared the highest number of genes, sharing 31 of the curated genes. This number is followed by ALS-cognitive impairment with 22, and finally ALS-dementia and ALS-frontotemporal dementia with 19 and 15 genes, respectively.

Enriched gene ontologies included terms classified as biological processes, cellular components and molecular functions databases. Among biological processes, the most significant gene sets contained terms that closely relate to ALS pathophysiology, for example, neuron death, regulation of apoptosis, response to oxidative stress, post-transcriptional protein modification, microglial activation, axonal transport, mitochondrial organization, vesicle mediated transport, autophagy regulation and ion transport. Furthermore, the most significant cellular component terms were related to axons, neuron projections, dendrites and vacuoles. Likewise, significantly enriched molecular functions included receptor binding, hydrolase activity, and growth factor activity.

### 3.4 Publicly available ALS curated gene lists and machine learning predictions overlap with our list of ALS-associated genes

To further assess the validity of our systematic review, we compared the list of ALS-associated genes with other curated lists found in the literature. Zhang et al. (2022) identified 690 ALS risk genes through regional fine-mapping (RefMap), a new machine learning method that integrates epigenetic profiling with GWAS summary statistics. Overall, they applied RefMap to ALS GWAS data, transcriptomic and epigenetic profiling of iPSC-derived motor neurons (MNs). Their ALS GWAS data used for RefMap gene enrichment included an independently curated list of genes (Eitan et al., 2022), and genes from the ClinVar database. With RefMap, they were able to identify ALS active genomic regions, which were mostly non-coding. They also determined that ALS pathogenesis is initiated in the distal axon of affected MNs. Finally, they established KANK1 as novel ALS gene, which is found in human neurons and leads to TDP-43 mislocalization (Neumann et al., 2006; Zhang et al., 2022). After manually comparing our list with theirs, we observed an overlap of 16 genes, including KANK1.

Eitan et al. (2022) performed a region-based burden analysis of variants in untranslated regions, including microRNAs (miRNAs), of ALS whole-genomes and non-ALS controls. They used whole-genome sequencing data from Project MinE ALS and NYCG ALS to analyze regions of interest. After performing the region-based burden test, where they combined rare genetic variants with minor allele frequencies (MAF) ≤ 0.01 to weigh their contribution to ALS, they identified 260 candidate genes associated to sporadic ALS. Overall, the strongest association found was for the untranslated region of IL18RAP, which was considered as a protective non-coding allele that reduces the chance of developing ALS five-fold and delays the onset in people who develop the disease. After comparing the three lists and eliminating duplicates, we observed that 29% (86/300) of our ALS-associated gene list has been reported in two independently curated lists of ALS-associated genes (Eitan et al., 2022; Zhang et al., 2022) as depicted in Figure 4C. Intriguingly, only 6 genes are found in the overlap between the three data sets. The lists of genes are included in Supplementary Table 8.

### 3.5 Drug-gene interactions analysis reveals hub genes

**FIGURE 5:**
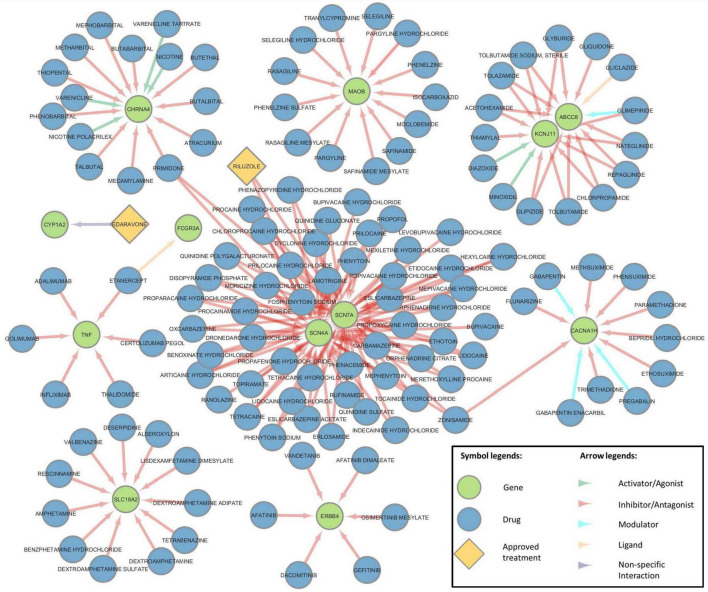
Drug-gene interaction network. Drug-gene interaction network depicting drugs (Blue, Yellow) targeting relevant ALS-associated genes (green) found in the systematic review. Hub genes with the highest number of targeting drugs are shown. Arrow colors indicate the interaction category.

The druggable genome consists of the group of genes that are known or predicted to interact with drugs in diverse conditions or disorders. To explore which of the ALS-associated genes found are part of the druggable genome, we used the DGIdb (Cannon et al., 2024) which contains over 10,000 genes and 20,000 drugs involved in nearly 70,000 drug-gene interactions. We identified a total of 2,836 drug-gene interactions involving 120 genes included in our systematic review. Among those, 357 interactions had a defined interaction type (inhibition, modulator, agonist, among others) and 2,479 had non-specific interactions. The full list of the retrieved interactions can be found in Supplementary Table 9. A drug-gene interaction network including only defined interactions and genes interacting with more than 2 drugs was created for visualization purposes (Figure 5). Even though non-specific, we added Edaravone and its interactor CYP1A2 into the network to overview both FDA-approved drugs for the treatment of ALS (Edaravone and Riluzole). Interestingly, the network analysis revealed that two genes that encode alpha subunit proteins of sodium channels, SCN4A and SCN7A, have the highest amount of drug interactions, and can also be inhibited by Riluzole.

SCN4A encodes for a voltage-gated sodium channel and SCN7A for a type II sodium channel whose activation is proportional to the extracellular sodium concentrations, thus mediating sodium homeostasis. These channels are essential for proper neuronal and muscular membrane depolarization during an action potential. There are two proposed mechanisms through which mutations in SCN4A can lead to an ALS phenotype. The first is through excessive sodium permeability leading to hyperexcitability and excitotoxicity or via retrograde motor neuron toxicity caused by muscular hyperexcitability (Franklin et al., 2020). SCN7A loss of function has been proposed to disrupt extracellular sodium homeostasis and lead to neuron hyperexcitability (Franklin et al., 2020).

The main therapeutic action of Riluzole is through the down-regulation of glutamic acid neurotransmission leading to a diminished neuronal excitotoxicity. Part of this effect is due to the inhibition of glutamate release from presynaptic dendrites, which may be down-regulated by the drug’s role as a modulator of voltage-gated sodium channels (Doble, 1996; Mohammadi et al., 2002; Sever et al., 2022). The modulation on the propagation of action potentials indirectly diminishes glutamate exocytosis, hence, excitotoxicity. Riluzole has been described to act on a variety of sodium channels and their subunits (Song et al., 1997; Weiss et al., 2010), including SCN7A and SCN4A, which are also reported on The ChEMBL Bioactivity Database (Mendez et al., 2019). Whether the effect of Riluzole on mutant SCN7A and SCN4A is intact, has not yet been studied. There are two case reports of patients with SCN4A mutations who were treated with Riluzole and died less than 2 years after symptom onset (Franklin et al., 2020). It is possible that Riluzole’s effectivity may be partially compromised in patients that are known carriers of mutations in these genes.

Interestingly, three of the genes with the most common and penetrant ALS mutations known (TARDBP, FUS, and SOD1) depict drug targets, however, their interaction type is not categorized and thus they are not shown in the network (Figure 5). An outstanding example are the variants of SOD1 which are targeted by Tofersen, an antisense oligonucleotide (Miller et al., 2022) Tofersen, a treatment designed specifically for SOD1-associated ALS was approved in April 2023 by the FDA after clinical trials (Miller et al., 2020, 2022; Benatar et al., 2022). Tofersen, being an antisense oligonucleotide, works by binding the mutant SOD1 transcripts and reducing their synthesis (Weishaupt et al., 2024).

Among the genes with interactions classified as “non-specific,” CYP1A2 was found to be targeted by Edaravone, an FDA approved drug for ALS treatment. Edaravone’s mechanism of action is still unclear, however, it is described as a reactive oxygen species scavenger. It is thought to trap free radicals and increase the expression of nuclear factor-erythroid factor 2 related factor (NrF2) which activates antioxidant response genes, thus protecting cells from ferroptosis (Homma) (Johnson et al., 2022; Soares et al., 2023). Even though Edaravone has been broadly approved for ALS population, its main benefit is in patients with definite or probable ALS either with milder symptoms (scoring at least 2 points in each of the items in the ALSFRS-R) or a disease duration less than 2 years (Soares et al., 2023).

### 3.6 Transcriptional dysregulation is observed in ALS public databases

**FIGURE 6:**
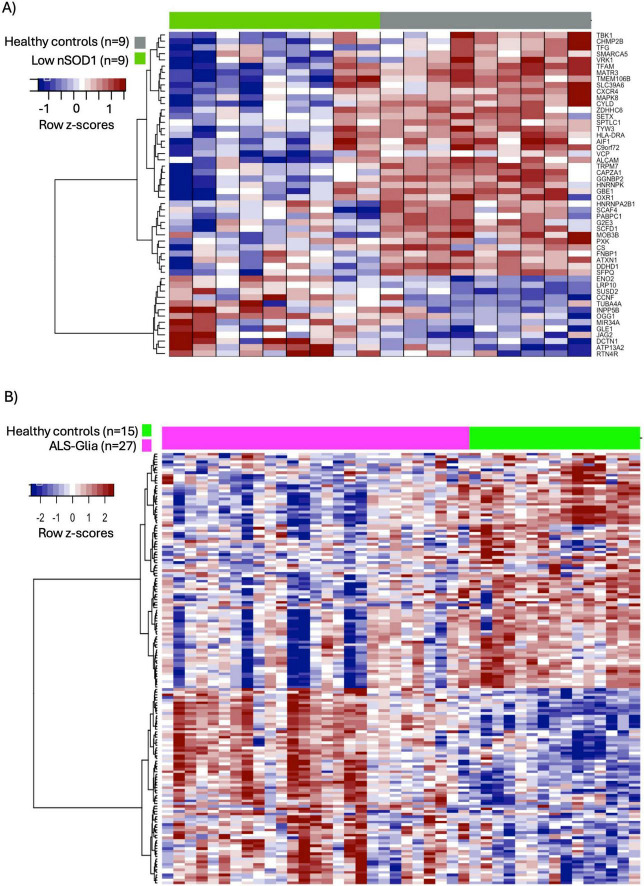
Heatmaps of differentially expressed ALS-associated genes in public ALS databases. The differential expression of ALS-associated genes was evaluated in **(A)** peripheral blood mononuclear cells (GSE183204) (Garofalo et al., 2022) and **(B)** postmortem cortex samples (GSE124439) (Tam et al., 2019). Raw count matrices were log-transformed and normalized. Genes were considered differentially expressed with fold-change > 1.1 and FDR < 0.1. Gene expression is depicted as row-z-scores.

To evaluate the transcriptional dysregulation of ALS-associated genes, we downloaded two ALS and neurologically healthy controls datasets corresponding to peripheral blood mononuclear cells (GSE183204) and postmortem cortex samples (GSE124439). Raw count matrices were log-transformed and normalized, and outlier samples were identified using PCA plots depicting 1,000 most variable genes. In the first dataset (GSE183204), consisting of peripheral blood mononuclear cells of ALS patients with low levels of nuclear SOD1 and age and sex-matched healthy controls (Garofalo et al., 2022), we identified four outlier samples (3 controls and 1 ALS). The remaining 9 ALS samples and 9 controls were compared. We focused on the list of ALS-associated genes and found 52 differentially expressed genes using Limma (Ritchie et al., 2015) with fold-change > 1.1 and FDR < 0.1. The heatmap of Figure 6A shows the row z-scores of the 52 differentially expressed genes which clearly separate ALS cases from healthy controls. As observed, the majority of ALS-associated genes are downregulated (39 down vs. 13 up). The top 5 most downregulated genes include CXCR4, CYLD, HLA-DRA, MATR3, and C9orf72.

**FIGURE 7:**
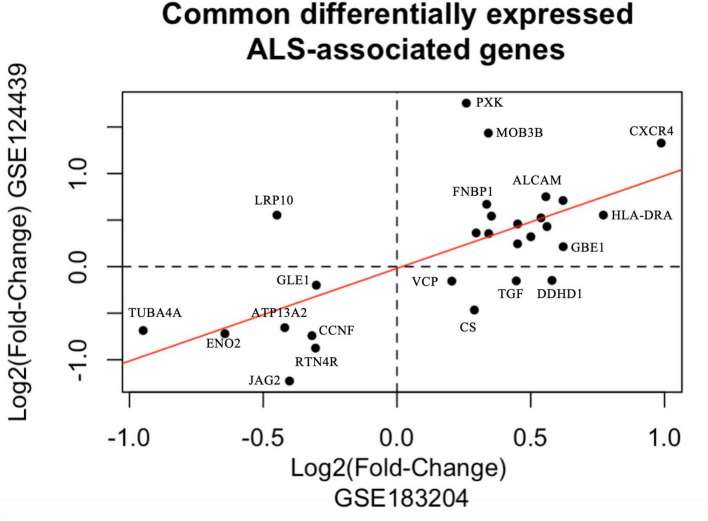
Comparison of differentially expressed ALS-associated genes. Comparison of log2-fold changes of differentially expressed ALS-associated genes found in two public databases (GSE183204) (Garofalo et al., 2022), (GSE124439) (Tam et al., 2019). Axes represent log2-fold changes. The regression line is depicted in red.

Similarly, we identified and filtered three outlier samples in the postmortem cortex dataset (GSE124439) corresponding to two controls and one ALS sample. The remaining 27 ALS samples, classified as activated microglia by the authors (Tam et al., 2019), were compared against 15 neurologically healthy controls using DESeq2 (Love et al., 2014). We found 163 differentially expressed genes with a fold-change > 1.1 and FDR < 0.1 (Figure 6B). From these genes, 89 were downregulated and 74 upregulated in ALS samples, suggesting a more robust dysregulation in postmortem cortex tissue than in peripheral blood mononuclear cells. The top 5 differentially expressed genes are upregulated and they include PXK, MOBP, SH3TC2, MBP, and MOB3B. Interestingly, Myelin Basic Protein (MBP) and Myelin-Associated Oligodendrocyte Basic Protein (MOBP) are both involved in oligodendrocyte-driven myelination whereas SH3 domain and tetratricopeptide repeats-containing protein 2 (SH3TC2) is thought to be expressed in the Schwann cells that wrap the myelin sheath around nerves (Stendel et al., 2010; Barateiro et al., 2016). These results support the proposition that oligodendrocyte dysfunction and myelin damage contribute to neuronal death in ALS (Kang et al., 2013). The molecular role of PXK and MOB3B in the pathophysiology of ALS is not clear. However, they are involved in cellular processes related to synaptic transmission, neuroprotection, and maintenance of cellular integrity (Mao et al., 2005; Takeuchi et al., 2010; Elkholi et al., 2023). The matrices of normalized counts and differential expression metrics of the expression of ALS-associated genes in the peripheral blood mononuclear cell and postmortem cortex samples are included in Supplementary Table 10. As observed in Figure 7, 28 ALS-associated genes are differentially expressed in both databases, depicting 23 genes with a significant fold-change in the same direction. See list of common differentially expressed ALS-associated genes and their fold-changes in each database in Supplementary Table 10.

## 4 Discussion

In ALS, pathological processes arise mainly from toxic gain-of-function, loss-of-function or toxic aggregates of proteins derived from variations/mutations in genes. Until recently, the number of ALS genes was only 40 (Chia et al., 2018; Goutman et al., 2018) and isolated efforts have been performed to increase this number using multi-omics and machine-learning approaches (Eitan et al., 2022; Zhang et al., 2022). Hitherto, we performed a systematic review to identify genes with variants associated with ALS. We comprehensively analyzed, filtered, and classified genes from 4,293 abstracts retrieved from PubMed, 7,343 variants from ClinVar, and 33 study accessions from the GWAS catalog. Our analysis yielded 300 genes with 479 ALS-associated variants. These genes were classified according to their association with ALS and the highest number of them were assigned to the experimentally validated group. Furthermore, other genes with ALS variants were classified as having GWAS evidence or suggested as a biomarker. As expected, the genes with the highest number of related publications are those with the most common and penetrant ALS mutations known: C9orf72, TARDBP, FUS, and SOD1 (Goutman et al., 2018). Moreover, the top 20 genes with the highest number of publications depict mainly pathogenic variants. Interestingly, 71% of ALS-associated variants are found in protein-coding regions (exons, TSS, and TTS), as opposed to what has been reported describing that most disease-associated variants are in non-coding regions (Smigielski et al., 2000; Hindorff et al., 2009). Furthermore, our results show that ALS associated genes are largely protein-coding with enzymatic activity. Although our aim was to capture both coding and noncoding variants associated with ALS, our search strategy was optimized for sensitivity to gene-linked evidence, thereby prioritizing variants with established gene associations. As a result, non-coding variants located in intergenic or enhancer regions without annotated gene links were excluded. While this may have introduced a gene-centric bias, the lack of gene annotation in these regions meant their inclusion would not have impacted our downstream analyses. Still, these non-coding elements remain underexplored and may play a more significant role in ALS pathogenesis as has been suggested (Cooper-Knock et al., 2021; van Rheenen et al., 2021). Moreover, to fully address the importance on non-coding variants, more whole-genome sequencing GWAS studies are needed.

We also investigated the molecular function and subcellular location of the proteins coded by our list of ALS-associated genes and found that 26% and 21% were found in the cytoplasm and in the nucleus, respectively, confirming that pathophysiological mechanisms of ALS include disturbed RNA metabolism, impaired autophagy/proteostasis, impaired DNA repair, and cytoskeletal defects (Nguyen et al., 2018). Moreover, we found significantly enriched gene sets relevant to ALS pathophysiology using our list of ALS-associated genes and we observed genetic overlap between other neurodegenerative disorders; for example, depression, cognitive impairment, frontotemporal dementia and dementia. This genetic overlap underlies the shared disease symptoms and supports the difficulty in their diagnosis.

We found that 29% of our list of ALS-associated genes has been reported in a manually curated and a machine-learning predicted list. This results likely reflect distinct biological dimensions of ALS, with literature-supported annotation versus data-driven functional predictions. The minimal convergence observed across gene sets suggests that machine learning and transcriptomic methodologies, while powerful, may not yet capture the full spectrum of ALS-relevant genes recognized in the literature, or are designed to selectively target rare genetic regions.

To analyze potential drugs for repurposing, we explored the drug-gene interactions using our list of ALS-associated genes. We retrieved a list of potential drugs targeting ALS-associated genes, including commonly used drugs (Riluzole and Edaravone) and recently FDA-approved drugs (Tofersen). Tofersen is a novel category of drugs consisting of antisense oligonucleotides that bind transcripts originating from genes with specific variants therefore reducing the synthesis of the mutant protein and thus decreasing its toxic effects. It is important to mention that these drugs only provide benefit in individuals carrying the specific gene variants, highlighting the importance of genetic screening and GWAS studies. Moreover, an increasing number of antisense oligonucleotide genetic drugs are being developed (Crooke et al., 2021), thus it is important to compile comprehensive catalogs of variants and genes associated with ALS.

We hypothesized that ALS-associated genes would depict transcriptional dysregulation and to verify it, we downloaded two public ALS data sets corresponding to samples from peripheral blood mononuclear cells and postmortem cortex (Tam et al., 2019; Garofalo et al., 2022). We performed a differential expression analysis and found that 17% and 54% of our list of 300 ALS-associated genes were significantly dysregulated in peripheral blood mononuclear cell and postmortem cortex samples, respectively. Interestingly, even though the fold-changes were considerably higher in the postmortem cortex samples, 28 genes were differentially expressed in both data sets, with 23 of them depicting dysregulation in the same direction. These results suggest that transcriptional dysregulation is not only observed in neurons, but also in mononuclear cells, thus, these transcripts are potential biomarkers of ALS. Among the common ALS-associated dysregulated genes, we found PXK, MOB3B, and CXCR4. Intriguingly, while the role of PXK and MOB3B in ALS remains elusive, researchers have demonstrated that they are involved in synaptic signaling and axonal survival and maintenance (Mao et al., 2005; Elkholi et al., 2023). Similarly, increasing evidence is implicating the CXC chemokines/cognate receptors signaling axes in the pathophysiology of ALS, suggesting that monitoring CXC-ligands (e.g. CXCR4) in ALS is important for tracking disease progression (La Cognata et al., 2024).

Several limitations in ALS genetic research should be highlighted. One significant challenge is the discrepancy between the number of ALS-related genes identified in PubMed compared to ClinVar or GWAS databases. This suggests a need for more comprehensive genetic studies and better registration practices, particularly in ClinVar, to ensure clinical significance and facilitate the application of findings. Furthermore, the heterogeneity in study design and data analysis complicates the integration of findings across studies, underscoring the importance of standardized methodologies and reporting practices. Another critical limitation is the lack of reproducibility across independent cohorts, often influenced by differences in sample size, geographic regions, and genetic backgrounds. Addressing these issues is vital for advancing ALS genetic research. Finally, there remains a significant gap in studying underrepresented ethnic populations. Most genetic research in ALS has focused on populations of European descent, leaving many other ethnic groups largely unexplored. Expanding genetic studies to include diverse populations is critical to gaining a comprehensive understanding of ALS pathogenesis and addressing disparities in disease outcomes.

Overall, this systematic review consolidates data from multiple databases to create a comprehensive catalog of genes and variants associated with ALS, presenting promising candidates for further validation studies. Our findings emphasize the need to transition from genetic associations to larger, more diverse case-control and cohort studies to deepen our understanding of ALS pathogenesis. Additionally, registering these variants in databases like ClinVar can enhance their utility in clinical and research contexts.

## Data Availability

The original contributions presented in the study are included in the article/Supplementary material, further inquiries can be directed to the corresponding authors.

## Footnotes

1
https://www.ncbi.nlm.nih.gov/clinvar/

2
https://www.ebi.ac.uk/gwas/home

3
https://www.gencodegenes.org/

## Supplementary material

Supplementary Figure 1 Schematic diagram depicting the retrieval and selection of ALS-variants associated to genes. Variants were compiled from ClinVar, PubMed, and GWAS Catalog databases while performing the systematic review. This figure is related to Figure 1.

Supplementary Table 1 List of publications retrieved from PubMed using the defined search query.

Supplementary Table 2 Genes retrieved through the systematic revision including their inclusion and exclusion categories.

Supplementary Table 3 Sentences from abstracts critical for assigning a gene’s category as annotated in PubTerm’s notes.

Supplementary Table 4 Collection of variants found in ALS-associated genes in GWAS and ClinVar databases.

Supplementary Table 5 Catalog of SNP identifiers compiled from PubMed, ClinVar, and GWAS databases through a systematic revision.

Supplementary Table 6 Classification of ALS-associated genes corresponding to gene type, subcellular location data, and TF function.

Supplementary Table 7 Gene set enrichment analysis using ALS-associated genes.

Supplementary Table 8 Comparison of ALS-associated genes to a manually curated list of ALS genes and a machine learning driven approach.

Supplementary Table 9 Table of drug-gene interactions retrieved from DGIdb (Cannon et al., 2024).

Supplementary Table 10 Differential gene expression analysis metrics of ALS-associated genes in public databases of peripheral blood mononuclear cells and postmortem cortex samples.
