# Supplementary figures and images for: A systematic review and functional in-silico analysis of genes and variants associated with amyotrophic lateral sclerosis
Source: Front Neurosci. 2025 Jun 16;19:1598336. doi: 10.3389/fnins.2025.1598336 (PMC12206810; doi:10.3389/fnins.2025.1598336)

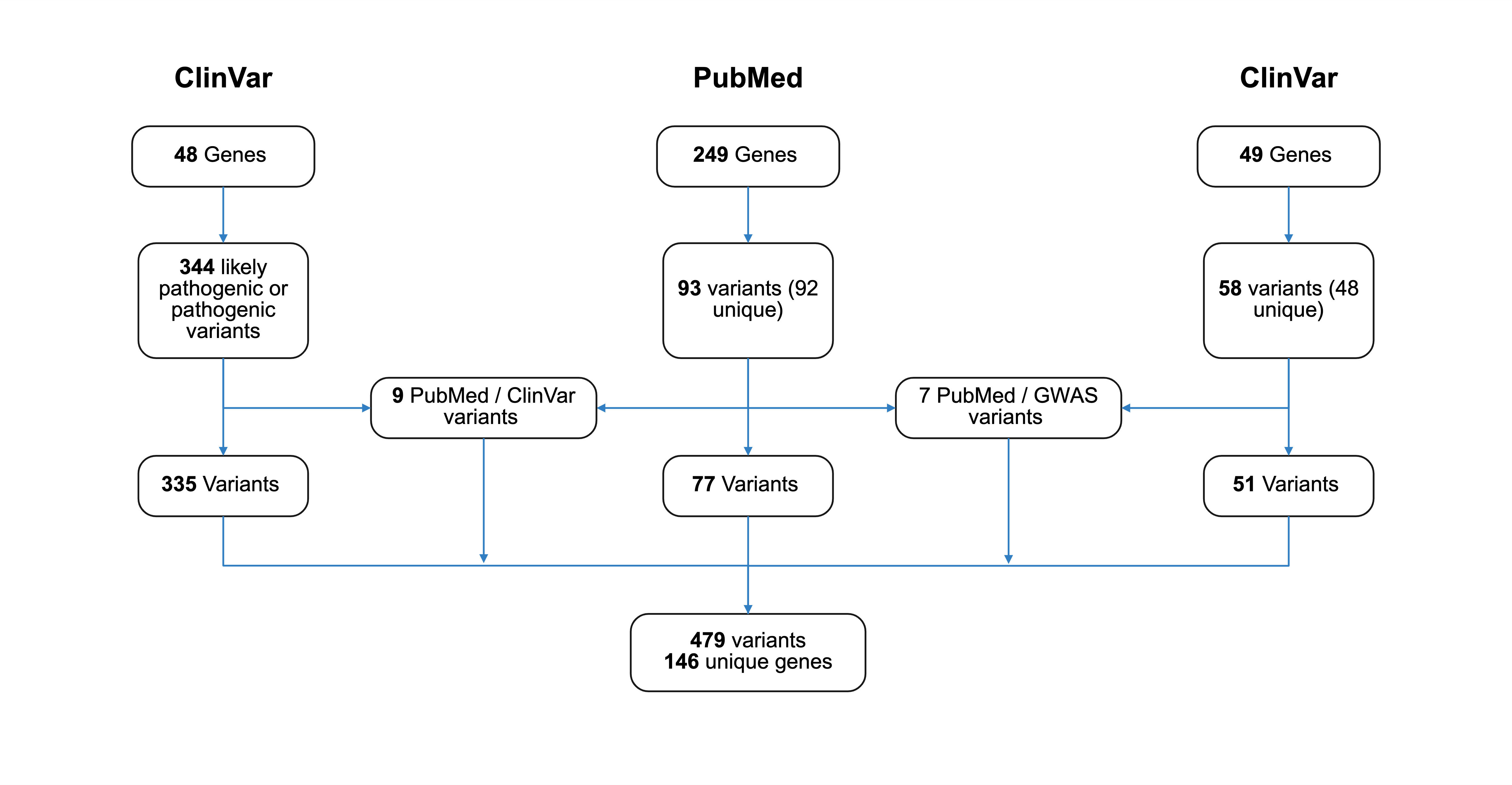

Supplement: Supplementary Figure 1 — Schematic diagram depicting the retrieval and selection of ALS-variants associated to genes. Variants were compiled from ClinVar, PubMed, and GWAS Catalog databases while performing the systematic review. This figure is related to Figure 1. [file Image_1.jpeg]
